# Sjögren’s disease patients with no hyposalivation and with short oral dryness complaints contain more T cells in salivary gland parenchyma

**DOI:** 10.1093/rheumatology/keag445

**Published:** 2026-08-20

**Authors:** Ting Yang, Silvia C Liefers, Bert van der Vegt, Kornelis S M van der Geest, Karina de Leeuw, Hendrika Bootsma, Frans G M Kroese, Sarah Pringle

**Affiliations:** Rheumatology and Clinical Immunology, University of Groningen, University Medical Centre Groningen, Groningen, The Netherlands; Rheumatology and Clinical Immunology, University of Groningen, University Medical Centre Groningen, Groningen, The Netherlands; Pathology and Medical Biology, University of Groningen, University Medical Centre Groningen, Groningen, The Netherlands; Rheumatology and Clinical Immunology, University of Groningen, University Medical Centre Groningen, Groningen, The Netherlands; Rheumatology and Clinical Immunology, University of Groningen, University Medical Centre Groningen, Groningen, The Netherlands; Rheumatology and Clinical Immunology, University of Groningen, University Medical Centre Groningen, Groningen, The Netherlands; Rheumatology and Clinical Immunology, University of Groningen, University Medical Centre Groningen, Groningen, The Netherlands; Rheumatology and Clinical Immunology, University of Groningen, University Medical Centre Groningen, Groningen, The Netherlands

**Keywords:** Sjögren’s disease, CD8^+^ T cells, salivary glands, hyposalivation, autoimmune disease

## Abstract

**Objective:**

Ninety-five percent of patients with the Sjögren’s disease (SjD) experience hyposalivation, which studies suggest is not strongly associated with salivary gland (SG) lymphocytic infiltration. Resident CD8^+^ T cells have recently been associated with SG pathology in SjD. We investigated the relationship between CD4^+^ T cell, CD8^+^ T cell, NK cell and macrophages number in the parenchyma of SjD patient SGs, and SG dysfunction.

**Methods:**

Quantification of CD4^+^ T cells, CD8^+^ T cells, NK cells and macrophages was performed using immunohistochemistry in parenchyma areas of SSA^+^ SjD patient parotid SGs with normal or reduced unstimulated and stimulated whole saliva (UWS, SWS), and different durations of subjective dryness complaints. Non-SjD sicca patients were included as controls.

**Results:**

We observed a significant increase in CD4^+^ T-cell (0.63%) and CD8^+^ T-cell frequency (0.77%) in parenchyma of SjD SGs compared with sicca controls (0.20% and 0.29%, respectively). NK and macrophages proportions did not differ significantly. A trend for increase in CD4^+^ and CD8^+^ T-cell frequency in SjD patient SGs with healthy levels of UWS and SWS flow, and with shortest oral dryness complaints, was observed compared with those with established flow rate reduction and longer dryness complaints. CD69 expression analysis suggests that CD4^+^/CD8^+^ T-cell populations may be derived from a mixture of tissue resident and infiltrated cells.

**Conclusion:**

Our data imply that the pre-SG disease stage in SjD is associated with an increase in CD4^+^ and CD8^+^ T cells in saliva-producing tissue areas.

Rheumatology key messages

- T-cell number increases in parenchyma of Sjögren’s disease (SjD) salivary glands compared with sicca controls.
- SjD patients without objective hyposalivation contain more CD8^+^ and CD4^+^ T cells in salivary gland parenchyma.

## Introduction

Hyposalivation (lack of salivary gland function) in the autoimmune condition Sjögren’s disease (SjD) has been appreciated since the initial description of the disease in 1933. Lack of salivary gland (SG) function imparts a dramatic decrease in patient quality of life. Similarly, the presence of lymphocytic infiltration in the SGs of SjD patients has also been long acknowledged, and is often presumed to directly cause SG dysfunction; however, several studies have demonstrated weak correlations between the degree of lymphocytic infiltration and SG dysfunction [1–3]. We and others have suggested that the reason for persistent SG hypofunction in SjD may be centred around dysfunctional salivary gland progenitor cells [4, 5]. Recent studies have implicated CD8^+^ T cells in dysfunction of SGs of SjD patients, but not necessarily in initiation of dysfunction [6–8]. In order to probe a potential role for CD8^+^ cells in initiation of SG dysfunction, we sought to perform a basic quantification study, examining the numbers of CD8^+^ T cells in saliva-producing areas (acinar cells plus interwoven saliva channelling intercalated ducts, ‘parenchyma’), in the parotid SGs of SSA^+^ SjD patients fulfilling the 2016 ACR EULAR criteria. We also included analyses of other known SG immune cell subsets (CD4^+^ T cells, NK cells and macrophages), used clinical data regarding saliva production to further examine the cell counts and included a sicca patient group as control.

## Patients and methods

### Patients

Parotid SG samples from both SjD and sicca controls were obtained during routine diagnostic evaluation for SjD. Patients were classified as having SjD if they fulfilled the 2016 ACR/ EULAR criteria. Institutional Review Board approval was obtained, and all patients provided informed consent (‘DiagnoSS cohort’, 202200009 and METc 2013.066). Clinical details can be found in Table 1. Focus score was defined by a trained pathologist.

**Table 1:** Patient information.

Patient characteristics	Sicca control	SjD SG group	
		UWS ≥0.1 ml/min	UWS <0.1 ml/min
*n*	7	10	10
Age (years), median (IQR)	46.50 (43.75–51.00)	57.00 (52.50–65.00)	61.00 (56.50–71.00)
Female, *n* (%)	7/7 (100.00)	7/10 (70.00)	9/10 (90.00)
Schirmer’s test result ≤5 mm/5 min, *n* (%)	1/7 (14.29)	6/10 (60.00)	5/10 (50.00)
Ocular staining score ≥5, *n* (%)	1/7 (14.29)	0/10 (0.00)	0/10 (0.00)
Median UWS (ml/min), median (IQR)	0.48 (0.25–0.69)	0.24 (0.21–0.36)	0.07 (0.02–0.08)
UWS ≤0.1 ml/min, *n* (%)	1/7 (14.29)	0/10 (0.00)	10/10 (100.00)
Median SWS (ml/min), median (IQR)	0.94 (0.54–1.36)	0.91 (0.78–1.61)	0.38 (0.28–0.72)
SWS ≤0.7 ml/min, *n* (%)	0/10 (0.00)	1/10 (10.00)	8/10 (80.00)
ACR/EULAR score, median (IQR)	1.00 (0.00–3.00)	4.00 (4.00–6.00)	5.00 (5.00–5.00)
Disease duration (diagnosis year)	N/A	3.00 (0.00–7.00)	5.50 (1.00–20.00)
FS, median (IQR)	0.00 (0.00–0.09)	0.76 (0.35–1.17)	0.15 (0.00–0.45)
FS positive, *n* (%)	0/7 (0.00)	4/10 (40.00)	1/10 (10.00)
SSA positive, *n* (%)	3/7 (42.86)	10/10 (100.00)	10/10 (100.00)

2016 ACR/EULAR score is the classification criteria for primary SS established by the ACR/EULAR in 2016. FS: focus score; IQR: interquartile range; N/A: not applicable; SG: salivary gland; SjD: Sjögren’s disease; SWS: stimulated whole saliva; UWS: unstimulated whole saliva.

### Immunohistochemistry

Automated immunohistochemistry was performed using the Benchmark automated staining platform (Ventana Medical Systems, Inc.). Immunostaining was performed on formalin-fixed, paraffin-embedded serial sections of tissue for the markers CD4 (Clone SP35, Ventana, CD4 T**-**cell marker, 1:20 dilution), CD8 (Clone C8/144B, DAKO, pre-diluted, for detection of CD8 T cells but with potential overlap with NK cells) and CD56 clone (MRQ-42, Ventana, pre-diluted) primarily associated with NK cells and macrophages. CD69 immunostaining, associated with tissue resident CD4^+^ and CD8^+^ T cells was performed manually (10803-1-AP, Proteintech, 1:600 dilution). All stained slides were digitized using a Philips UFS slide scanner (Philips, Best, The Netherlands) and assessed using Philips IntelliSite Pathology Solution software.

In Visiopharm (2023.01) an algorithm was developed and used to detect areas of tissue containing saliva-producing acinar cells. Considering that intercalated ducts are interwoven with acinar cells, our algorithm for detection of acinar cells will also contain these cells. The algorithm was based on the classification method deep learning, using the DeepLabv3+ network, and trained for 248K iterations, until it properly outlined acini/intercalated ductal cell areas. Fat cells, fibrotic cells and striated and excretory ductal cells were excluded. For ease of reading and the purpose of this study, we refer to this acinar cell plus intercalated duct area as the ‘parenchyma’. Within the parenchyma area, including any immune cells, the number of nuclei were counted using the Visiopharm Nuclei Detection, AI (Hoersholm, Denmark). Manual counting of CD4, CD8, CD56 and CD69 stained cells was performed blinded and separately for each staining on three manually selected, matched parenchyma areas of ∼2.21 mm^2^ in total, per marker protein, per patient.

### Statistics

Comparisons between two groups were performed using independent samples *t*-tests. Homogeneity of variance was assessed using Levene’s test, and Welch’s correction was applied when equal variance assumptions were not met. For comparisons among multiple groups, differences in the proportions of CD4^+^ T cells, CD8^+^ T cells, CD69^+^ cells, macrophages and NK cells were analysed using two-way analysis of variance. Tukey’s honestly significant difference post-hoc test was applied for data with homogeneous variances, and Games–Howell multiple comparisons testing was used for data with unequal variances.

## Results

### CD4^+^ and CD8^+^ T cells increase in abundance in parenchyma of SjD salivary glands compared with sicca controls

**Figure 1:**
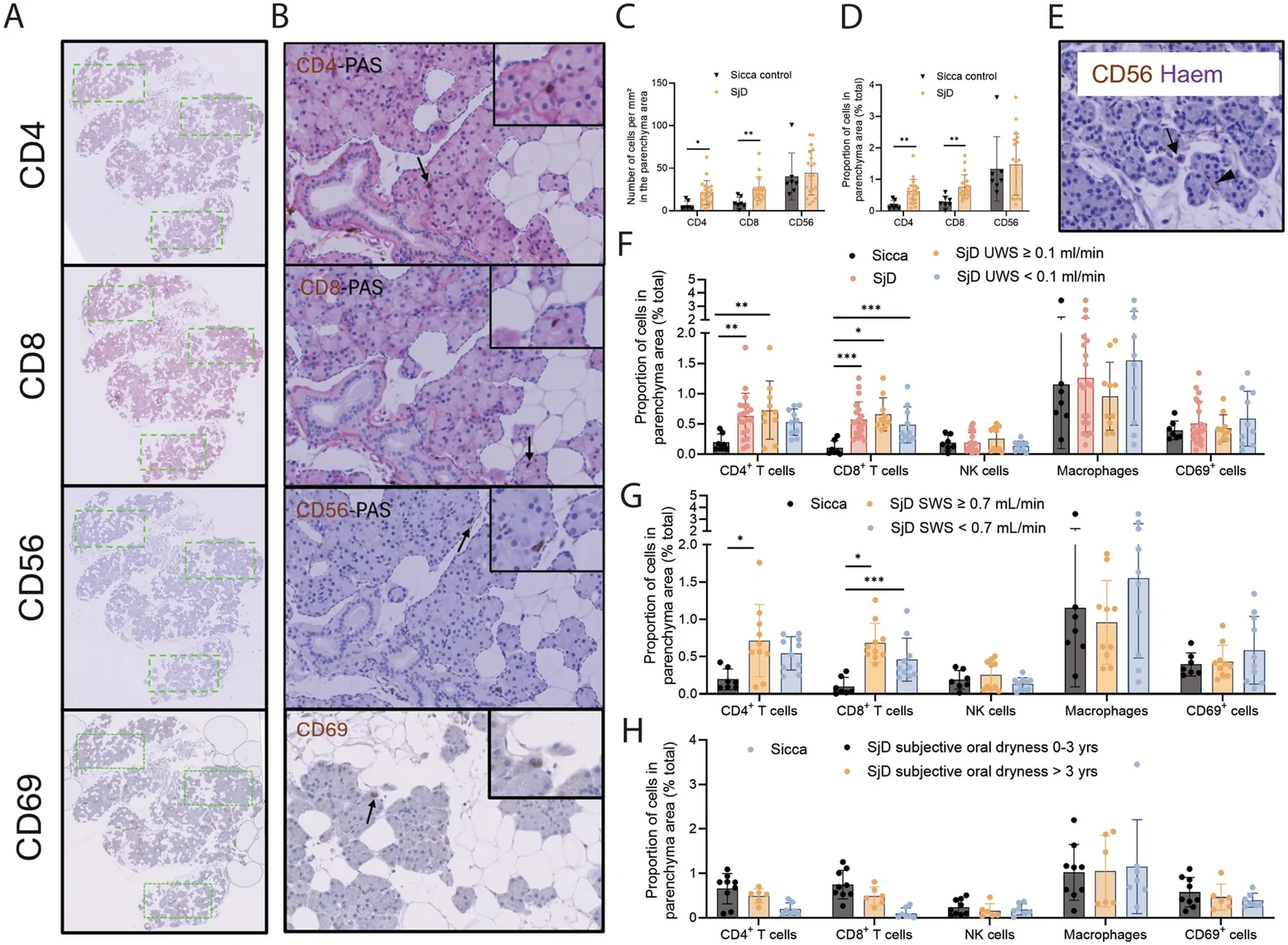
SjD patients without objective hyposalivation and with shorter duration of oral dryness complaints show a trend for more CD4^+^ and CD8^+^ T cells in SG parenchyma. (**A**) Serial sections showed matched areas analysed. (**B**) Examples of CD4-PAS, CD8-PAS, CD56 and CD69 immunostaining. (**C**) CD4, CD8 and CD56 cell counts per mm^2^ of tissue. *N* = 7 for sicca controls, 20 for SjD patients. (**D**) Proportions of CD8^+^, CD4^+^ and CD56⁺ cells within selected parenchymal areas of SG sections across groups; *n* = 7 sicca control patients and 20 SjD patients. (**E**) Example of CD56 immunostaining in parenchyma of a salivary gland of a SjD patient. Elongated macrophage-like cell marked with a long arrow, while round NK-like cells are marked with short arrow. (**F**) Proportion of CD4^+^ T cells, CD8^+^ T cells, NK cells, macrophages and CD69^+^ cells in acinar areas of sicca control and SjD patient parotid SGs; *n* = 7 sicca controls and 20 for SjD, 10 for each for UWS subgroup. (**G**) As for (E), but with SjD stratification based on SWS cut-off of 0.7 ml/min. (**H**) As for (E), but with SjD stratification based on numbers of years of oral dryness complaints (0–3 or >3 years). In (F–H), bars represent s.d. Asterisks represent statistical significance between indicated groups. *P < 0.05, **P < 0.01, ***P < 0.001. PAS: periodic acid–Schiff; SG: salivary gland; SjD: Sjögren’s disease

Three areas of parenchyma per tissue of 2.2 mm^2^ were selected, per tissue section per immunostaining. These areas contained a broadly consistent number of total cells (acinar + intercalated ducts + immune cells), with a mean of 7442 cells ± 1095 SD per 2.2 mm^2^ tissue, reflecting the relatively homogeneous nature of parenchymal areas both between tissue of the same patient and between patients (Supplementary Fig. S1A). Example serial section strategy and immunostainings for CD4, CD8 CD56 and CD69 are shown in Fig. 1A and B.

Initial CD4^+^, CD8^+^ and CD56^+^ cell numbers per mm^2^ tissue in sicca controls and all SjD patients grouped together are shown in Fig. 1C, whereby significantly more CD4^+^ cells (21.4/mm^2^) and CD8^+^ cells (25.4/mm^2^) were detected in the total SjD patient groups, compared with sicca controls (6.3/mm^2^ CD4^+^ cells and 9.4/mm^2^ CD8^+^ cells; Fig. 1C, *P* < 0.05 and *P* < 0.01, respectively). When applying our parenchymal nuclei quantification to the areas, our enumeration of CD4^+^ and CD8^+^ cells (number of CD4^+^ and CD8^+^ relative to all nuclei in parenchyma area) slightly altered, whereby we observed a stronger increase in CD4 expressing cells in parenchyma areas of SjD patients (0.63%) when comparing with sicca controls (0.20%; *P* < 0.01 Fig. 1D), compared with that in Fig. 1C. The difference in CD8^+^ T-cell number remained significantly different between SjD patients (0.78%) and sicca controls when our parenchyma algorithm was applied (0.29%, *P* < 0.01; Fig. 1D).

SGs also contain tissue resident macrophages and NK cells, in addition to CD4^+^ and CD8^+^ T cells. CD56 expression can be used to highlight macrophages and/or NK cells. In our initial count data, no significant difference in number of CD56 expressing cells was observed between sicca control and SjD groups (Fig. 1D). CD8^+^ T cells, NK cells and macrophages can be distinguished from each other more precisely using a combination of CD8 and CD56 expression, and CD56^+^ cell morphology (‘compact’ or ‘elongated’; Fig. 1E, Supplementary Fig. S1B and C). We performed this differentiation on our cell-count dataset. The proportion of likely CD8^+^ T cells, once corrected for potential overlap with NK/macrophages, increased more strikingly than the raw data (i.e. uncorrected counts), when comparing the sicca control group (0.10% ± 0.12 SD) with SjD patients (0.57% ± 0.29 SD, *P* < 0.001; Fig. 1F). The proportion of inferred macrophages and NK cells in parenchyma did not show any significant change of expression between sicca control and SjD patients (Fig. 1F).

In order to investigate whether the T cells we enumerated represent tissue resident cells (CD8 T_rms_) or CD4^+^ T cells (CD4 T_rms_), or infiltrating circulating cells, we performed immunostaining for the T_rm_ marker protein CD69. In sicca patients, the combined CD4^+^ T cell and CD8^+^ T-cell count roughly equalled that of the CD69^+^ cells, suggesting that most T cells were T_rm_ in nature (Supplementary Fig. S1D). In SjD SGs as a whole group, a greater number of CD4^+^ and CD8^+^ T cells (0.6% and 0.5% of parenchyma, respectively) was observed than CD69^+^ cells (0.5% of parenchyma), suggesting presence of recent emigrants of CD4^+^ and/or CD8^+^ T cells into the acinar tissue (Supplementary Fig. S1D).

### SjD patients without objective hyposalivation and with shorter subjective oral dryness complaints years show a trend for more CD8^+^ and CD4^+^ T cells in SG parenchyma

Since loss of saliva production in SjD does not correlate strongly with focus score, we reasoned that our study should not centre around grouping SjD patients based on differing focus scores. Instead, we chose to analyse SjD patient SGs stratified on basis of normal *vs* reduced saliva production and no/short duration of oral dryness complaints, compared with those with more established dryness. To that end, our dataset purposely included SjD patients fulfilling the ACR/EULAR criteria, but who do not demonstrate reduced unstimulated whole saliva (UWS) rate (>0.1 ml/min flow rate), stimulated whole saliva (SWS) rate (>0.7 ml/min flow rate) or record shorter (0–3 years) subjective reports of xerostomia, in an attempt to probe the ‘pre-SG disease’ stage stadium.

When our total pool of SjD patients was split by UWS cut-off of 0.1 ml/min, surprisingly, both CD4^+^ T cells (0.73%) and CD8^+^ T cells (0.69%) showed a trend for higher abundance in SjD patients with normal UWS flow rate (> 0.1 ml/min) compared with both SjD patients with UWS flow rate of ≤0.1 ml/min (0.53% and 0.48% respectively; Fig. 1F) and sicca control patients (0.20% and 0.10%). A similar trend was also observed when examining SWS with the cut-off of 0.7 ml/min for reduction of flow (Fig. 1G). In SjD, it is well established that subjective sicca complaints present years before disease diagnosis, suggesting that the processes triggering hyposalivation in SjD occur long before official diagnosis. Interestingly, a trend for higher number of CD4^+^ and CD8^+^ T cells was also observed in patients with shorter duration of subjective oral dryness complaints (Fig. 1H). In Fig. 1F–H, proportions of immune cells–stratified SjD groups remained higher than those of sicca controls at the group level, in UWS, SWS and dryness complaints measures. Stratification of the CD69^+^ cell population identified based on UWS, SWS or duration of oral complaints did not reveal any striking trends (Supplementary Fig. S1D), underpinning that the CD4^+^ and CD8^+^ T-cell population potentially responsible for loss of SG function may be a mixture of resident and infiltrating cells, although these data require further crystallization (Fig. 1F–H).

## Discussion

Our data suggests that frequencies of CD4^+^ and CD8^+^ T cells in saliva-producing parenchyma (acinar cells plus intercalated ducts in this study) increases in SGs from patients with SjD compared with sicca controls. Intriguingly, frequencies of CD4^+^ and CD8^+^ T-cell populations and combinations showed a trend for greater number in SjD patients with no objective reduction in UWS or SWS, and with shorter subjective complaints of oral dryness. In this study, we make a cautious assumption that the majority of SjD patients will development hyposalivation, and therefore that the increase in CD4^+^ and CD8^+^ T cells observed in SjD patients with normal UWS and SWS flow rates might represent a pre-SG dysfunction stage.

Our data would therefore suggest that tissue resident CD4^+^ and CD8^+^ T cells are activated and/or proliferative at a very early stage of SG pathology in SjD, and also the possibility that recent T-cell emigrants contribute to this pool.

Most studies focusing on the role of T cells in dysfunction of the SG in SjD centre around CD8^+^ T cells, and additionally at disease stages in which lymphocytic infiltration and loss of SG function is already established. For instance, Mauro *et al.* demonstrated that tissue-resident CD8^+^CD103^+^CD69^+^ T cells (T_rm_ cells) located in lymphocytic foci are significantly expanded in the SG of SjD patients compared with SG of non-SjD (sicca) patients, and exhibit cytotoxic and proinflammatory phenotypes, including high expression of granzyme B and IFN-γ [7]. Gao *et al.* reported thatCD8^+^ T cells with a CD69^+^CD103^+^ T_rm_ phenotype outnumbered CD4^+^ T cells in the labial SGs of patients with SjD, and were both between salivary duct epithelial cells and acinar cells [6]. Pranzatelli *et al*. also demonstrated that immune infiltrates enriched in GZMK^+^ CD8^+^ T cells could be found in the SGs of SjD patients. The authors further suggest that these T cells drive type I IFN signalling via induction of mitochondrial dysfunction, and subsequent activation of the cGAS-STING pathway in a particular population of PRR4^+^CST3^+^WFDC2^−^ acinar cells [8]. Taken together, we can conclude that granzyme and/or IFNγ-expressing T cells may have capabilities to trigger SG dysfunction, and in theory that CD4^+^ T cells, which are also capable of producing IFNγ, could also do this. Thus, a greater number of T cells, as demonstrated in our study, before a measurable drop in saliva production may represent a biologically significant turning point in the SG in SjD, whereby a threshold of acinar cell damage is reached. This damage may impart both a drop in saliva production, and the instigation of an inflammatory milieu. Whether CD4^+^ T cells can also trigger acinar dysfunction via expression of other cytokines such (IL-17, IL-21) will be the subject of future interesting studies.

Our data beg the question of T cells’ mechanism of activation, as well as their potential role in SG dysfunction. Plausible options for activation, based on existing literature, include local inflammation and epithelial (auto)antigen presentation. In terms of the former, many studies have linked viral infections with SjD, although their absolute causative nature has never been verified [9]. In terms of the epithelial (auto)antigens, factors such oxidative or metabolic stress, or the presence of IFNs, may enhance the ability of SG epithelial cells to function as non-professional antigen-presenting cells. In this capability, their exposure to SjD-associated auto-antigens (such as Ro/SSA and La/SSB), normally located intracellularly, on the outer surface of the cell membrane in association with class I and class II MHC molecules, may promote activation of SSA- or SSB-specific CD8^+^ or CD4^+^ T cells, respectively [10–13]. A recent single-cell analysis of (CD4 and CD8) TCR repertoires in SSA^+^ SjD patients with presence of lymphocytic infiltration revealed was not able to identify target antigens, leaving this question over antigen-dependency still very much open [14].

Limitations are as follows. Our study represents a preliminary, simple immunohistochemical study, which is easily applicable, but limited in the interpretations we can make. Future directions including multiplex SG immunostaining of this interesting SjD patient cohort with normal saliva production would add more depth of information to our hypothesis, as of course would transcriptomics studies. Our sicca control group also contained some patients who were SSA^+^. Comparison of SjD CD4 and CD8 T-cell number with an entirely SSA–sicca group may strengthen our findings further.

In summary, we cautiously propose that CD4^+^ and CD8^+^ T cells are salient to initiation of SG dysfunction in SjD. The mechanisms triggering their activation and/or influx remain to be elucidated.

## Supplementary Material

keag445_Supplementary_Data

## Data Availability

The data underlying this article are available in the article.
