# Supplementary material for: Sjögren’s disease patients with no hyposalivation and with short oral dryness complaints contain more T cells in salivary gland parenchyma
Source: Rheumatology (Oxford). 2026 Aug 20;65(9):keag445. doi: 10.1093/rheumatology/keag445 (PMC13541375; doi:10.1093/rheumatology/keag445)
Supplement: Supplementary file 1 [file keag445_supplementary_data.docx]

Yang et al_Supplementary data

**Figure S1**. A) Hematoxylin stained parotid salivary gland tissue (upper panel) following machine learning for detection of acinar cell areas, outlined in blue dashed line (middle panel) and subsequent analysis for number of nuclei present in acinar cell area (lower panel). B) Table summarizing differences in CD8 and CD56 marker protein expression and morphology, between CD8^+^ T cells, NK cells and Macrophages. C) Schematic representation of method employed to enumerate CD8^+^ T cells, NK cells and macrophages. , N.D. = no data; not a likely cell population. D) Quantification of the number of CD4+ T cells, CD8+ T cells and CD69+ cells in sicca control and SJD patients. N= 7 for sicca controls, and 20 for SjD group.
